# Physical Activity Protects Against the Negative Impact of Coronavirus Fear on Adolescent Mental Health and Well-Being During the COVID-19 Pandemic

**DOI:** 10.3389/fpsyg.2021.580511

**Published:** 2021-03-11

**Authors:** Laura J. Wright, Sarah E. Williams, Jet J. C. S. Veldhuijzen van Zanten

**Affiliations:** School of Sport, Exercise and Rehabilitation Sciences, College of Life and Environmental Sciences, University of Birmingham, Birmingham, United Kingdom

**Keywords:** anxiety, COVID-19, exercise, fatigue, stress, youth

## Abstract

**Background:** The severity of the Coronavirus pandemic has led to lockdowns in different countries to reduce the spread of the infection. These lockdown restrictions are likely to be detrimental to mental health and well-being in adolescents. Physical activity can be beneficial for mental health and well-being; however, research has yet to examine associations between adolescent physical activity and mental health and well-being during lockdown.

**Purpose:** Examine the effects of adolescent perceived Coronavirus prevalence and fear on mental health and well-being and investigate the extent to which physical activity can be a protective factor against these concerns.

**Methods:** During United Kingdom lockdown restrictions, 165 participants (100 female, aged 13–19) completed an online questionnaire assessing perceived Coronavirus prevalence and fear, physical activity, and indicators of mental health and well-being (stress, anxiety, depression, fatigue, vitality, and perceived health). Separate hierarchical multiple linear regression analyses (with age, gender, perceived Coronavirus prevalence, and fear entered in step 1, and physical activity in step 2) were run to predict each well-being outcome.

**Results:** Regression analyses indicated that in general, while Coronavirus fear was a negative predictor, physical activity was a positive and stronger predictor of enhanced mental health and well-being outcomes.

**Conclusion:** Findings suggest that physical activity during the Coronavirus pandemic can counteract the negative effects of Coronavirus fear on adolescent mental health and well-being. Therefore, physical activity should be promoted during lockdown to support good mental health and well-being.

## Introduction

The first cases of the COVID-19 Coronavirus (SARS-CoV-2) were discovered in Wuhan Province of China at the end of December 2019, and by 11th March 2020, the WHO declared this new Coronavirus a pandemic (World Health Organization, 2020). To combat the spread of the virus, unprecedented regulations were put in place for people displaying symptoms (self-isolation) or having been in contact with people with symptoms (quarantine), and most countries applied community-wide restrictions on movement and daily activities (commonly referred to as lockdowns). For example, during the United Kingdom lockdown, people were only allowed to leave the house for basic necessities (e.g., seeking medical attention and food shopping), to go to work if this was essential and could not be done from home, and to exercise once a day (UK Government, 2020). While these measures were deemed necessary to limit the spread of the Coronavirus, such restrictions are likely to have a negative impact on mental health.

Quarantine and larger scale lockdowns led to separation from loved ones, fear over the health of oneself, family, and friends, and a need to cope with the new situation (Cava et al., 2005). All these things can influence mental health and well-being. Indeed, research shows that restrictions during previous epidemics and pandemics have led to increased stress (and even post-traumatic stress disorder), depression, anxiety, emotional exhaustion, and fear (Brooks et al., 2020; Jiménez-Pavón et al., 2020; Xiang et al., 2020). Individuals also experienced numerous stress-evoking factors during lockdown and quarantine such as fear of infection and death, loss of social contacts, confinement, helplessness, as well as experienced stress, depression, anxiety, panic attacks, and even suicidality (Brooks et al., 2020; Jiménez-Pavón et al., 2020; Xiang et al., 2020). Emerging studies relating to the COVID-19 Coronavirus pandemic observe similar patterns, in that large proportions of healthcare workers and the general public surveyed have reported symptoms of depression, anxiety, and distress (Huang and Zhao, 2020; Lai et al., 2020; Rodríguez-Rey et al., 2020).

Specifically, fears and concerns surrounding pandemics during lockdowns and quarantine (e.g., fear of infection and fear of becoming ill) can have a particularly negative impact on mental health and well-being. Research shows that fear of becoming ill was evident in over 20% of people who had been quarantined during severe acute respiratory syndrome (SARS) epidemic (Reynolds et al., 2008). In line with this, perceived severity of the current Coronavirus, as well as being in close contact with someone diagnosed with the Coronavirus have both been found to be associated with increased stress, anxiety, and depression in adults (Rodríguez-Rey et al., 2020). Furthermore, fear of the current Coronavirus was associated with higher levels of anxiety in the general population (Harper et al., 2020). This research suggests that concerns about the current Coronavirus are likely to be detrimental to mental health and well-being.

Most research relating to the COVID-19 Coronavirus has focused on adult populations, with less attention to adolescent populations. This is somewhat surprising given the high prevalence of mental health problems in adolescents (Sadler et al., 2018). Furthermore, lockdown restrictions leading to sudden school closures, a switch to online and more independent learning, and the cancelation of end of year final exams were all likely to contribute to stress and anxiety. Adolescents were also confined to home, organized sports, and group physical activity stopped and they were no longer able to socialize in person with others outside their household. It is, therefore, not surprising that initial Coronavirus pandemic research in adolescents found a high prevalence of depressive and anxiety symptoms (Chen et al., 2020; Zhou et al., 2020). Therefore, it is important that effective strategies are identified to promote mental health and well-being to protect adolescents against the negative effects of the Coronavirus.

One effective way to enhance mental health and well-being is physical activity. More physically active individuals exhibit lower levels of stress, anxiety, depression, and fatigue as well as greater vitality and well-being in adults and adolescents (Petruzzello et al., 1991; O’Connor and Puetz, 2005; Stults-Kolehmainen and Sinha, 2014; Biddle et al., 2019; Rodriguez-Ayllon et al., 2019; Gianfredi et al., 2020). Physical activity can have a beneficial effect, equal to or greater than, a range of common mental health treatments, such as cognitive behavioral therapy for anxiety (Wipfli et al., 2008), and is comparable to antidepressant medication for depression (Dinas et al., 2011). Importantly, it is not only more physical activity that can improve mental health. A sudden decrease in physical activity can negatively impact on depressive symptoms, anxiety, fatigue, and energy levels (Weinstein et al., 2017). In sum, there is ample evidence that being and staying physically active results in benefits for mental health and well-being.

Previous research suggests that during the Coronavirus pandemic, physical activity could contribute to better mental health and well-being in adolescents. Moreover, physical activity’s importance has been acknowledged by governments in several countries, by allowing physical activity to be one of the few reasons people could leave their homes during lockdown. Despite this, lockdown restrictions make physical activity more challenging, with research suggesting reduction in adult physical activity levels during the Coronavirus compared to pre-Coronavirus (Ammar et al., 2020). However, research conducted during the Coronavirus pandemic on adults has shown that physical activity is related to better mental health, such as lower levels of depression, stress, and anxiety (Rodríguez-Rey et al., 2020). Additionally, self-reported reductions in physical activity since the onset of the Coronavirus have been associated with higher stress, depression, and anxiety (Stanton et al., 2020). Conversely, in a sample of less physically active individuals, those who report engaging in more physical activity during lockdown restrictions report lower anxiety than those who report being less physically active during lockdown (Lesser and Nienhuis, 2020).

When investigating physical activity, mental health, and well-being, it is important to take gender differences into consideration. For example, there is evidence that males are more physically active than females (Bann et al., 2019). Furthermore, females tend to report higher levels of anxiety, depression, and stress than their male counterparts (Murray et al., 2011; Sadler et al., 2018). In addition, in the context of Coronavirus, there is evidence for higher stress and anxiety levels in females (Fitzpatrick et al., 2020; Lai et al., 2020; Mazza et al., 2020). Consequently, when examining the associations between physical activity and mental health and well-being, it is important to investigate the impact on gender.

In sum, although there is substantial evidence of the detrimental effects of quarantine and lockdown on mental health and well-being, less is known about the factors impacting mental health and well-being in adolescents. While concerns related to the Coronavirus may adversely affect mental health, physical activity is likely to be beneficial. However, this is yet to be examined in an adolescent population. Therefore, the present study aimed to examine the effects of Coronavirus concerns on mental health and well-being in adolescents and the extent to which physical activity can protect against the negative impact of these Coronavirus concerns on mental health and well-being during lockdown. A secondary aim was to investigate the effect of gender on these variables.

## Materials And Methods

### Participants

In total 100 female and 65 male participants aged between 13 and 19 years old (*M* = 15.90, *SD* = 1.48) took part in the study, 94.6% identified their ethnicity as white. Participants were recruited predominately through emails to schools, sports clubs, and other organizations targeted at adolescents (e.g., Scouts) across the United Kingdom. The study was also advertised *via* the social media of the research team. All participants lived in England. None of the participants reported as having tested positive for COVID-19. The study obtained ethical approval from the University ethics committee, and all participants, and where appropriate, a parent/guardian, provided informed consent. Power analyses showed that a sample of 165 participants would allow for the detection of a small to medium effect with power at 0.90 (Faul et al., 2009).

### Procedures

Data collection took place from 1st May 2020 to the 25th May 2020 in the United Kingdom using an online questionnaire platform (SmartSurvey). Participants then completed an online questionnaire pack including the demographic information, and measures to assess perceived fear and prevalence of the Coronavirus, physical activity, perceived stress, anxiety, depression, fatigue, vitality, and general health. Coronavirus restrictions and school closures had been in place for 5 weeks at the onset of data collection and remained unchanged during data collection.

### Questionnaires

All questionnaires were phrased so that the time period they related to was during the last month, which coincided with the period of the United Kingdom lockdown restrictions.

#### Coronavirus Inventory

The Coronavirus inventory was developed for the present study by modifying the Swine Flu Inventory (SFI) items (Wheaton et al., 2012). The SFI is a 10-item questionnaire which assessed things such as individuals’ concerns about the spread of H1N1 influenza, perceptions of likelihood of contracting the infection, and severity of the infection. Items were modified in the present study to refer to the Coronavirus that causes COVID-19 rather than H1N1 influenza, and any reference to the United States was altered to refer to the United Kingdom (e.g., “How quickly do you believe contamination from Swine Flu is spreading in the U.S.?” was modified to “How quickly do you believe the Coronavirus is spreading in the U.K.?”). Small wording modifications were also made to ensure that items were understandable by an adolescent population. Two further items “to what extent has the threat of the Coronavirus influenced your well-being” and “to what extent has the threat of the Coronavirus increased your stress levels” in order to better tap some of our variables of interest, namely stress and well-being and how they relate to the threat of the Coronavirus (see Supplementary Table S1 for all items). Participants rated the extent to which they agreed with each item on a 5-point Likert scale ranging from 0 (very little) to 4 (very much).

To reduce the items to a number of meaningful factors, principle axis factoring with oblimin rotation was conducted on the 12 items (Tabachnick and Fidell, 2013). The initial solution identified four factors with eigenvalues ranging from 1.02 to 3.92, collectively accounting for 63.67% of the variance. However, one item (item 6) failed to load onto any factor and one item (item eight) cross loaded highly on more than one factor. These two items were removed from the second iteration which revealed three factors. The third factor consisted of only two items which poorly loaded on their subscale (items 9 and 10). These two items were dropped for the third run which yielded a final two factor solution with eigenvalues of 1.34 and 3.25, accounting for 57.36% of the variance. Each factor consisted of four items and factor loadings were all above 0.45 (Tabachnick and Fidell, 2013). One factor contained items assessing perceived prevalence and likelihood of becoming infected with the Coronavirus (e.g., “How likely do you think it is that you could become infected with the Coronavirus?”). This subscale was named *perceived Coronavirus prevalence*. The other factor contained items tapping concerns about the coronavirus and impact it could have on health and well-being (e.g., “If you did become infected with the Coronavirus, to what extent are you concerned that you will be severely ill?”). This subscale was named *Coronavirus fear*. The final eight items and their factor loadings are reported in Table 1, Cronbach alpha coefficient values demonstrated good reliability for Coronavirus concerns (0.78) and slightly low reliability for perceived Coronavirus prevalence (0.68). Mean scores for each subscale were calculated, so a higher score indicated a greater perceived Coronavirus prevalence or Coronavirus fear.

**Table 1:** Coronavirus inventory factor loadings for a two-factor solution.

Item	Coronavirus fear	Coronavirus prevalence
To what extent has the threat of the Coronavirus increased your stress levels?	0.817	
To what extent are you concerned about the Coronavirus?	0.690	
To what extent has the threat of the Coronavirus influenced your well-being?	0.679	
If you did become infected with the Coronavirus, to what extent are you concerned that you will be severely ill?	0.483	
How quickly do you believe the Coronavirus is spreading in the United Kingdom?		0.634
To what extent do you believe that the Coronavirus is prevalent in the United Kingdom?		0.601
How likely do you think it is that you could become infected with the Coronavirus?		0.546
How likely is it that someone you know could become infected with the Coronavirus?		0.495

#### Physical Activity

Physical activity was measured using a single item in which participants selected which level represented their physical activity (Jurca et al., 2005). The question asked participants to rate their usual pattern of activity. In the present study, this was altered to ask participants their usual pattern of activity in the last month. Participants selected one of five possible levels with each increasing level indicating a higher amount of physical activity [Level 1: “Inactive or little activity other than usual daily activities”; Level 2: “Regularly (≥5 days/week) participate in physical activities requiring low levels of exertion that result in slight increases in breathing and heart rate for at least 10 min at a time”; Level 3–5: participation in “aerobic exercises such as brisk walking, jogging, or running at a comfortable pace, or other activities requiring similar levels of exertion” for 20–60 min per week (level 3), 1–3 h per week (level 4) or over 3 h per week (level 5)]. The item has been used as an element of a non-exercise estimate of cardio-respiratory fitness (CRF) which was found to be a good estimation of CRF when compared to actual exercise testing (Jurca et al., 2005). Single item physical activity measures have been found to provide reliable and valid assessments of physical activity in adolescents (Scott et al., 2015).

#### Perceived Stress Scale

The 10-item Perceived Stress Scale (PSS; Cohen et al., 1983) assessed how stressed individuals felt over the past month. Participants read each item (e.g., “How often have you felt nervous and ‘stressed’?”) and respond on a 5-point Likert scale from 0 (never) to 4 (very often). Positively worded items are reverse scored, and a mean score is calculated of all items so that a higher score indicates a higher level of perceived stress. The PSS has been reported to have good internal reliability in adolescent populations (Carlozzi et al., 2010). The present study demonstrated good internal reliability (*α* = 0.88).

#### Hospital Anxiety and Depression Scale

The Hospital Anxiety and Depression Scale (HADS; Zigmond and Snaith, 1983) assessed general levels of anxiety (seven items, e.g., “I get sudden feelings of panic”) and depressive symptoms (seven items, e.g., “I feel as if I am slowed down”), with items being scored on a scale of 0–3. Each subscale is summed with scores ranging from 0 to 21, with higher scores indicating higher levels of anxiety or depressive symptoms. The HADS has been validated for use in adolescents to assess anxiety and depressive symptoms (White et al., 1999). The present study demonstrated good internal reliability for anxiety (*α* = 0.84) and depression (*α* = 0.81).

#### Multidimensional Fatigue Inventory

The 20-item Multidimensional Fatigue Inventory (MFI-20; Smets et al., 1995) was used to measure general fatigue (e.g., “I feel tired”), physical fatigue (e.g., “Physically I feel only able to do a little”), reduced activity (e.g., “I get little done”), mental fatigue (e.g., “It takes a lot of effort to concentrate on things”), and reduced motivation (e.g., “I do not feel like doing anything”). Each subscale consisted of four items. Participants rated the extent to which they agree or disagree with each item on a 5-point Likert scale from 1 (no, that is not true) to 5 (yes, that is true). Positively worded items were reverse scored and scores for each subscale were summed to create a total score for each subscale with higher scores representing greater fatigue. The MFI-20 is a valid and reliable measure to assess fatigue (Smets et al., 1995), used successfully in adolescent populations (Vantieghem et al., 2018). The present study demonstrated good internal reliability for all subscales (*α* ≥ 0.75).

#### Subjective Vitality Scale

The Subjective Vitality Scale (SVS; Ryan and Frederick, 1997) is a 5-item questionnaire (e.g., “I feel I have a lot of energy”) assessing how energetic a person feels. Participants rate the extent to which they agree with each statement on a 7-point Likert scale from 1 (not at all true) to 7 (very true). Items are then averaged with higher scores indicative of greater vitality. The SVS has been found to be a valid and reliable measure of vitality in adolescents (Reinboth et al., 2004). The current study demonstrated a good internal reliability (*α* = 0.89).

#### General Health

Perceptions of general health was measured using a single item taken from the Short Form Health Survey-12 (Ware et al., 1996). Participants rated their perceived health on a 5-point scale: poor, fair, good, very good, and excellent. This single item measure for perceived health has been found to be as valid and reliable as a multi-item scale (Macias et al., 2015) and has also been used effectively in adolescents (Herman et al., 2015).

### Data Analysis

All data were analyzed using SPSS (IBM, Version 26). First, data were screened and cleaned to check for missing data and outliers. As Little’s MCAR test showed that less than 5% of data were missing at random (*p* > 0.05), the expectation maximization method was used to complete the data set (Tabachnick and Fidell, 2013). Inspection of the Mahalanobis distance at *p* < 0.001 identified no multivariate outliers so all data were retained for the analysis. Descriptive statistics were generated for males and females and a series of one-way ANOVAs conducted to examine gender differences in all relevant outcomes. Pearson correlations were then run to examine the relationships between perceived Coronavirus prevalence, Coronavirus fear, and physical activity, with each other, as well as the mental health and well-being outcomes (i.e., perceived stress, anxiety, depressive symptoms, different fatigue subscales, vitality, and perceived general health). Finally, separate hierarchical multiple linear regressions were run to predict each well-being outcome. To determine the extent to which perceived Coronavirus prevalence and Coronavirus fear predicted each well-being outcome, these two variables were entered in step 1 along with control variables gender and age. Then physical activity was added at step 2 to determine if it was an independent predictor of each mental health and well-being outcome. The alpha level was set at 0.05 for all analyses and standardized beta values are reported for all regression analyses.

## Results

### Participant Characteristics

Means and SDs of perceived Coronavirus prevalence, Coronavirus fear, physical activity, and mental health and well-being outcomes for both males and females are reported in Table 2. One-way ANOVAs revealed that males reported significantly lower Coronavirus fear, perceived stress, anxiety, general fatigue, physical fatigue, and mental fatigue, as well as higher vitality and general health. There was no significant difference in physical activity levels between male and female participants.

**Table 2:** Mean (SD) of perceived Coronavirus prevalence, Coronavirus fear, physical activity, and well-being outcomes for male and female participants.

	Male mean (SD)	Female mean (SD)	*F* value	*p* value
Perceived Coronavirus prevalence	2.39 (0.876)	2.57 (0.67)	2.17	0.143
Coronavirus fear	1.62 (0.96)	2.19 (0.97)	14.44	<0.001
Physical activity	3.41 (1.35)	3.42 (1.3)	<0.01	0.958
Perceived stress	1.85 (0.691)	2.32 (0.67)	18.73	<0.001
Anxiety	6.62 (3.55)	9.35 (4.71)	16.13	<0.001
Depressive symptoms	5.17 (3.78)	6.27 (3.92)	3.23	0.074
Vitality	3.94 (1.40)	3.28 (1.32)	9.66	0.002
General health	3.44 (0.906)	3.08 (1.13)	4.59	0.034
General fatigue	11.33 (3.28)	12.66 (3.96)	5.11	0.025
Physical fatigue	8.79 (3.53)	10.25 (3.91)	5.99	0.015
Reduced activity	11.67 (4.31)	11.88 (4.54)	0.09	0.763
Mental fatigue	12.33 (3.55)	13.92 (3.74)	7.45	0.007
Reduced motivation	11.52 (3.97)	12.39 (4.09)	1.87	0.173

Degrees of freedom: 1,164, F value relates to gender differences ANOVA.

### Correlational Analyses

Correlation analysis between perceived Coronavirus prevalence, Coronavirus fear, physical activity, and well-being outcomes are displayed in Table 3. Perceived Coronavirus prevalence was associated with higher levels of Coronavirus fear, but physical activity was not associated with either perceived Coronavirus prevalence or Coronavirus fear. Coronavirus fear was associated with higher levels of perceived stress, anxiety, depressive symptoms, general fatigue, reduced activity, mental fatigue, and reduced motivation, and was associated with lower levels of vitality and general health. Coronavirus prevalence was not associated with any of the well-being outcomes. Higher levels of physical activity were associated with lower levels of perceived stress, depressive symptoms, and all five fatigue subscales, as well as higher levels of vitality and perceived general health.

**Table 3:** Correlations between perceived Coronavirus prevalence, Coronavirus fear, physical activity and well-being outcomes.

	Perceived Coronavirus prevalence	Coronavirus fear	Physical activity
Perceived Coronavirus prevalence	1		
Coronavirus fear	0.41^**^	1	
Physical activity	0.01	−0.09	1
Perceived stress	0.10	0.40^**^	−0.26^**^
Anxiety	0.11	0.47^**^	−0.15
Depressive symptoms	−0.10	0.17^*^	−0.31^**^
Vitality	0.02	−0.18^*^	0.34^**^
General health	−0.05	−0.25^**^	0.33^**^
General fatigue	0.07	0.24^**^	−0.38^**^
Physical fatigue	0.11	0.25^**^	−0.58^**^
Reduced activity	<−0.01	0.07	−0.54^**^
Mental fatigue	0.01	0.20^**^	−0.23^**^
Reduced motivation	0.09	0.19^*^	−0.32^**^

* *p* < 0.05.

** *p* < 0.001.

### Multiple Linear Regressions

Results of the multiple linear regression analyses are reported in Tables 4 and 5. Step 1 of these analyses explored the independent associations between Coronavirus fear and perceived coronavirus prevalence with the different measures of mental health and well-being, while correcting for age and gender, with physical activity being added as a predictor at step 2. Results for all regressions, except when predicting depressive symptoms, showed that perceived Coronavirus prevalence was not independently associated with any mental health and well-being outcomes.

**Table 4:** Regressions between perceived Coronavirus prevalence and fear, physical activity, stress, anxiety, depressive symptoms, vitality, and general health.

	Perceived stress		Anxiety		Depressive symptoms		Vitality		General health	
Step 1	ΔR^2^ = 0.23, F = 11.72^a^, *p* < 0.001		ΔR^2‑^ = 0.27, F = 14.59^a^, *p* < 0.001		ΔR^2^ = 0.08, F = 3.35^a^, *p* = 0.012		ΔR^2^ = 0.11, F = 4.89^a^, *p* = 0.001		ΔR^2^ = 0.09, F = 4.18^a^, *p* = 0.003	
	ß	p	ß	p	ß	p	ß	p	ß	p
Gender	0.25	0.001	0.19	0.010	0.11	0.187	−0.23	0.004	−0.13	0.114
Age	0.13	0.071	0.05	0.504	0.06	0.445	−0.18	0.021	−0.14	0.059
Perceived Coronavirus prevalence	−0.09	0.263	−0.11	0.136	−0.21	0.014	0.12	0.144	0.08	0.366
Coronavirus fear	0.36	0.000	0.47	<0.001	0.23	0.009	−0.16	0.068	−0.24	0.006
Step 2	ΔR^2^ = 0.05, F = 10.55^b^, *p* = 0.001		ΔR^2^ = 0.01, F = 2.50^b^, *p* = 0.12		ΔR^2^ = 0.08, F = 15.87^b^, *p* < 0.001		ΔR^2^ = 0.10, F = 20.62^b^, *p* < 0.001		ΔR^2^ = 0.09, F = 17.20^b^, *p* < 0.001	
Gender	0.25	0.001	0.19	0.009	0.11	0.142	−0.24	0.002	−0.13	0.080
Age	0.11	0.096	0.04	0.564	0.04	0.575	−0.16	0.030	−0.13	0.083
Perceived Coronavirus prevalence	−0.07	0.340	−0.10	0.162	−0.19	0.020	0.10	0.203	0.06	0.485
Coronavirus fear	0.33	0.000	0.45	<0.001	0.19	0.022	−0.12	0.154	−0.20	0.016
Physical activity	−0.22	0.001	−0.11	0.116	−0.29	<0.001	0.32	<0.001	0.30	<0.001

a Degrees of freedom are 4,161.

b Degrees of freedom are 1,160.

**Table 5:** Regressions between perceived Coronavirus prevalence and fear, physical activity, and fatigue.

	General fatigue		Physical fatigue		Reduced activity		Mental fatigue		Reduced motivation	
Step 1	ΔR^2^ = 0.10, F = 4.50^a^, *p* = 0.002		ΔR^2^ = 0.09, F = 3.86^a^, *p* = 0.005		ΔR^2^ = 0.04, F = 1.66^a^, *p* = 0.16		ΔR^2^ = 0.07, F = 3.21^a^, *p* = 0.015		ΔR^2^ = 0.04, F = 1.75^a^, *p* = 0.14	
	ß	p	ß	p	ß	p	ß	p	ß	p
Gender	0.14	0.074	0.14	0.072	0.04	0.670	0.17	0.034	0.07	0.418
Age	0.17	0.023	0.10	0.173	0.19	0.018	0.05	0.550	0.06	0.476
Coronavirus prevalence	−0.04	0.601	<−0.01	0.984	−0.05	0.569	−0.09	0.292	0.01	0.902
Coronavirus fear	0.22	0.012	0.21	0.017	0.07	0.417	0.19	0.030	0.16	0.064
Step 2	ΔR^2^ = 0.12, F = 25.36^b^, *p* < 0.001		ΔR = 0.31, F = 83.40^b^, *p* < 0.001		ΔR^2^ = 0.28, F = 65.15^b^, *p* < 0.001		ΔR^2^ = 0.04, F = 7.94^b^, *p* = 0.005		ΔR^2^ = 0.09, F = 17.02^b^, *p* < 0.001	
Gender	0.15	0.043	0.16	0.016	0.05	0.491	0.18	0.026	0.07	0.345
Age	0.15	0.032	0.07	0.258	0.16	0.021	0.03	0.659	0.04	0.617
Coronavirus prevalence	−0.02	0.798	−0.04	0.598	−0.01	0.853	−0.07	0.365	0.03	0.705
Coronavirus fear	0.17	0.034	0.14	0.055	<0.01	0.952	0.16	0.058	0.13	0.139
Physical activity	−0.35	<0.001	−0.56	<0.001	−0.22	<0.001	−0.21	0.005	−0.31	<0.001

a Degrees of freedom are 4,161.

b Degrees of freedom are 1,160.

Step 1 and step 2 of the regressions predicting stress, anxiety, and depressive symptoms accounted for a significant portion of the variance (except anxiety step 2). Coronavirus fear was a significant predictor in step 1 and step 2, with higher levels predicting more perceived stress, anxiety, and depressive symptoms. Physical activity was a significant negative predictor for stress and depressive symptoms, with higher levels of physical activity predicting lower levels of these outcomes.

The regressions for the fatigue subscales showed step 1 accounted for a significant portion of the variance and Coronavirus fear was a significant positive predictor of these subscales (with the exception of reduced activity and reduced motivation). Step 2 accounted for a significant proportion of the variance and physical activity was a significant negative predictor of all five fatigue subscales. Furthermore, Coronavirus fear became a nonsignificant predictor in all regressions except general fatigue.

Step 1 and step 2 of the regressions predicting vitality and general health accounted for a significant proportion of the variance. In step 1 and step 2, Coronavirus fear was a significant negative predictor of general health but not vitality. In step 2, physical activity was a positive predictor of both variables so that higher levels of physical activity were associated with greater vitality and perceived health.

## Discussion

This study aimed to examine the extent to which Coronavirus prevalence and Coronavirus fear predicted adolescents’ mental health and well-being during lockdown, and the extent to which physical activity can protect against the negative impact of these Coronavirus concerns on mental health and well-being. Despite perceived Coronavirus prevalence and fear being associated with each other, higher Coronavirus fear was associated with higher levels of stress, anxiety, depressive symptoms, and fatigue, as well as lower vitality and general health, which is in line with research in adults (Brooks et al., 2020; Harper et al., 2020; Rodríguez-Rey et al., 2020). Perceived Coronavirus prevalence was only significantly associated with lower depressive symptoms. The seemingly surprising negative relationship between perceived Coronavirus prevalence and depressive symptoms could perhaps be explained by behavioral disengagement (Horwitz et al., 2011): adolescents with higher depressive symptoms may not engage with media related to Coronavirus, making them less aware of its prevalence. Collectively, the findings demonstrate that Coronavirus fear is a more consistent predictor of poorer mental health compared to perceived Coronavirus prevalence. This is similar to research in adult populations demonstrating fear of the current Coronavirus to be associated with higher anxiety and depressive symptoms, and fear of infection during periods of quarantine from other viruses to be associated with stress (Brooks et al., 2020; Harper et al., 2020). By extending these relationships into other indicators of health and well-being, the results of the present study suggest that it is not the prevalence of the Coronavirus but rather the fear of the impact it could have on health which is associated with poorer adolescent mental health.

An important novel finding of the current study is that physical activity counteracted the negative impact of Coronavirus fear on mental health and well-being in adolescents. Moreover, the size of the beta weights in the regressions predicting depression, vitality, perceived health, and fatigue (particularly physical fatigue and reduced activity) demonstrate that physical activity was a stronger predictor than Coronavirus fear. Indeed, physical activity is suggested to impact mental health in different ways, and some of these pathways might be especially relevant during the Coronavirus pandemic (Mikkelsen et al., 2017; Matias et al., 2020). For example, physical activity can have an immediate positive effect on mood and feelings of energy (Liao et al., 2017) and physical activity can be a distraction from negative thoughts and stress related to the Coronavirus fear (Mikkelsen et al., 2017). Physical activity can also bring a structure or daily routine to life, which is likely to be heavily disturbed as a result of the lockdown. As such, it can provide a sense of control and mastery, which can also impact on well-being (Mikkelsen et al., 2017).

The associations between physical activity and more positive mental health and well-being may in part be due to the environment of the activity. Although physical activity location was not assessed in the current study, in other studies during the Coronavirus, outdoor physical activity has been reported in over 90% of individuals (Lesser and Nienhuis, 2020). During the data collection in the present study, physical activity was one of the few reasons adolescents could leave the house. Furthermore, data from May to July 2020 indicate that more adolescents were being active outdoors, with the number of people going for a walk and cycling for fun or fitness being higher compared with the same period in 2019 (Sport England, 2021). Consequently, it could be suggested that a number of the study participants may have been doing most of their physical activity outdoors. Outdoor physical activity is generally associated with lower depression, tension, anger, and confusion compared to indoor physical activity (Bowler et al., 2010; Dunton et al., 2015), and has been associated with improved emotional well-being, including in adolescents (Pasanen et al., 2014). Moreover, adults who spent more time doing physical activity outdoors during the current Coronavirus displayed better well-being (described as “flourishing”; Lesser and Nienhuis, 2020). Therefore, the simple act of being able to leave the confinements of the house to exercise may have had an additional benefit to mental health. However, data also suggest an increase in adolescent participation in gym and fitness during this same time compared with 2019 (Sport England, 2021). Considering that public gyms were closed as part of lockdown restrictions, it is likely that these gym and fitness activities were undertaken at home (e.g., live streamed workouts). Given that the location of the physical activity was not assessed in the present study, these suggestions are purely speculative.

Although physical activity significantly predicted lower perceived stress, Coronavirus fear was the strongest independent predictor of stress. This is not surprising given that literature shows infection fear can induce stress during periods of quarantine or lockdown (Brooks et al., 2020). A bi-directional relationship between stress and physical activity has been reported, with stress leading to less physical activity and less physical activity leading to more stress (Stults-Kolehmainen and Sinha, 2014). Importantly, the present study shows that despite the strong association between Coronavirus fear and perceived stress, physical activity was still a significant negative predictor of stress. Interestingly, during the SARS outbreak in Hong Kong in 2003, especially those who experienced more stress at home and worry about the virus reported to have increased their time spend being physically active (Lau et al., 2005, 2006). Therefore, it is possible that physical activity is also used by adolescents as a way to cope with the stress of the COVID-19 Coronavirus and to have a sense of control over their health.

A somewhat surprising result from the present study was that physical activity was not associated with anxiety. This contradicts work outside of pandemic settings, where higher levels of physical activity are associated with lower anxiety in children and adolescents (Ahn and Fedewa, 2011). During the current Coronavirus pandemic, the associations between physical activity and anxiety appear complex. Similar to the present study, other Coronavirus related studies have shown no association between anxiety and physical activity (Zhang et al., 2020), and no difference in anxiety between those who are active and non-active (Lesser and Nienhuis, 2020; Zhang et al., 2020). However, other studies have shown that those who are more physically active during the pandemic displayed lower levels of anxiety (Antunes et al., 2020). It is difficult to know why the relationship between physical activity and anxiety appears to be inconsistent during the current Coronavirus, but it may be that anxiety experienced during this time is different in nature to the anxiety typically experienced in the absence of such pandemics. During disease outbreaks, a number of people experience clinical levels of fear and anxiety (Taylor, 2019), and recent research suggests that individuals can experience specific dysfunctional Coronavirus anxiety (Lee et al., 2020). Although findings in the current study do not suggest specifically high levels of anxiety compared to non-pandemic situations, anxiety may be caused by other factors which influence its associations with physical activity. Other studies during the Coronavirus pandemic have shown that a reduction in physical activity compared to pre-Coronavirus is associated with higher anxiety, and inactive people who became more active during the Coronavirus displayed lower anxiety than those who become less active (Lesser and Nienhuis, 2020), suggesting that it could be the change in physical activity that is more closely associated with anxiety in such times. However, further research is needed to fully understand the relationship between physical activity and anxiety during lockdowns due to pandemics.

Coronavirus fear, perceived stress, and anxiety were all significantly higher in females, which is in line with Coronavirus research in adult populations (Fitzpatrick et al., 2020; Lai et al., 2020; Mazza et al., 2020). As adolescent females have been found to have higher levels of stress and anxiety (Murray et al., 2011; Sadler et al., 2018) under regular circumstances, it is perhaps expected that their levels would also be higher under a pandemic scenario. The present study suggests that females may also display greater fear associated with the Coronavirus. In addition, general, physical, and mental fatigue were higher, and vitality lower in females, which has been reported before in adults (Engberg et al., 2017), but not during the Coronavirus pandemic. To our knowledge, this is the first study to find this in adolescents. Interestingly, there was no gender difference in physical activity. This is perhaps surprising given that a number of studies report males displaying significantly higher levels of physical activity compared with females (Bann et al., 2019). However, other studies (albeit in college students) have also found no gender differences in certain types of physical activity during lockdown (Zhang et al., 2020). Thus, commonly reported gender differences in adolescent physical activity (Bann et al., 2019) could be influenced by lockdown restrictions – indeed, research suggests that most individuals report experiencing a reduction in physical activity compared to pre-lockdown (Stanton et al., 2020).

The findings of the study indicate the clear benefits of physical activity to help to counteract the negative mental implications of Coronavirus fear in adolescents. The data suggest positive benefits for mental health with physical activity in adolescents. United Kingdom physical activity guidelines recommend adolescents to participate in 60 min of moderate to vigorous physical activity per day (Department of Health and Social Care, 2019). Although the measure of physical activity used in the current study does not allow for comparison of levels of physical activity against the physical activity guidelines, it does confirm with general physical activity guidelines that being more physically active is associated with better (mental) health.

Despite its novel contributions, the study is not without limitations. First, the cross-sectional design cannot imply causation. The study also uses a self-report single item measure of physical activity; however, this method has been previously validated (Jurca et al., 2005; Scott et al., 2015). Third, our measure of Coronavirus prevalence and fear was designed for the purpose of the study and has thus not been extensively validated. However, items underwent exploratory factory analysis based on the recommendations in the literature and the results support the existence of two separate subscales (Tabachnick and Fidell, 2013). Finally, our sample’s ethnicity was predominately white. Given the reported ethnicity differences in risk of mortality relating to the Coronavirus (Price-Haywood et al., 2020), it would be interesting to explore these associations in a wider range of ethnicities.

In conclusion, the present study found that while Coronavirus fear was associated with higher levels of stress, anxiety, depressive symptoms, fatigue, and lower vitality and general health, physical activity was an independent predictor of lower stress, depressive symptoms, and fatigue, and higher vitality and perceived health. Moreover, physical activity was often a stronger predictor than Coronavirus fear. Therefore, this was the first study to show that physical activity during the Coronavirus pandemic can counteract the negative effects of fear of the Coronavirus on adolescent mental health and well-being. Findings highlight the significance of physical activity during the Coronavirus pandemic and emphasize the importance of governments letting individuals continue to leave the house for physical activity during periods of lockdown.

## Data Availability Statement

The raw data supporting the conclusions of this article will be made available by the authors, without undue reservation.

## Ethics Statement

The studies involving human participants were reviewed and approved by University of Birmingham Science, Technology, Engineering and Mathematics Ethical Review Committee. Written informed consent to participate in this study was provided by the participants’ legal guardian/next of kin.

## Author Contributions

All authors contributed to the design of the project, data collection, and wrote the manuscript.

### Conflict of Interest

The authors declare that the research was conducted in the absence of any commercial or financial relationships that could be construed as a potential conflict of interest.
