# Supplementary material for: Physical Activity Protects Against the Negative Impact of Coronavirus Fear on Adolescent Mental Health and Well-Being During the COVID-19 Pandemic
Source: Front Psychol. 2021 Mar 11;12:580511. doi: 10.3389/fpsyg.2021.580511 (PMC7990778; doi:10.3389/fpsyg.2021.580511)
Supplement: Supplementary file 1 [file Data_Sheet_1.pdf]

Supplementary Table 1: All items from the Coronavirus Inventory

**1**	**To what extent are you concerned about the Coronavirus?**
**2**	**To what extent do you believe the Coronavirus is prevalent in the UK?**
**3**	**How likely do you think it is that you could become infected with the Coronavirus?**
**4**	**How likely is it that someone you know could become infected with the Coronavirus?**
**5**	**How quickly do you believe the Coronavirus is spreading in the UK?**
6	How much exposure have you had to information about the Coronavirus?
**7**	**If you did become infected with the Coronavirus, to what extent are you concerned that you will be severely ill?**
8	To what extent has the threat of the Coronavirus influence your decision to be around people?
9	To what extent has the threat of the Coronavirus influenced your travel plans?
10	To what extent has the threat of the Coronavirus influenced your use of safety behaviours (e.g., hand sanitizer)?
**11**	**To what extent has the threat of the Coronavirus influenced your well-being?**
**12**	**To what extent has the threat of the Coronavirus increased your stress levels?**

Note: Items in bold are included in the final Coronavirus Inventory
